# Protective effect of *Zataria multiflora *Boiss against sodium nitrite-induced hepatotoxicity in rats 

**DOI:** 10.22038/AJP.2021.18781

**Published:** 2022

**Authors:** Fatemeh Ahmadi, Ali Louei Monfared, Neamatollah Shakarami

**Affiliations:** 1 *Department of Histology and Bacteriology, Faculty of Para Veterinary, Ilam University, Ilam, Iran*

**Keywords:** Z. multiflora Boiss, Histology, Sodium nitrite, Liver

## Abstract

**Objective:**

Sodium nitrite (NaNO_2_) is used as a color stabilizer and antimicrobial agent in preservation of cured meat and fish. However, extensive use of this agent in the meat industries increased worries about its detrimental effects on human health. *Zataria multiflora* (*Z. multiflora) *is a well-known plant with therapeutic properties in the traditional medicine**.** Therefore, the present study was conducted to investigate the protective effect of this plant against sodium nitrite-induced hepatotoxicity.

**Materials and methods:**

Thirty-two male Wistar rats were divided into 4 groups: Control (without any treatment), nitrite (350 mg/kg by gavage for 60 days), NaNO_2_ plus *Z. multiflora* (rats treated with NaNO_2_ 350 mg/kg gavage for 60 days and simultaneously received *Z. multiflora* extract at 200 mg/kg, ip) and *Z. multiflora* group (rats treated with *Z. multiflora* extract at 200 mg/kg, ip). At the end of the study, rats were euthanized and liver tissue samples were taken and studied under microscopy. Also, serum levels of liver function enzymes and antioxidant defense systems were measured. The results were analyzed using SPSS software and a p<0.0.5 was considered significant.

**Results:**

Results showed that NaNO_2 _induces liver injuries and altered hepatic histo-architecture. Also, NaNO_2 _significantly altered the biochemical profiles and antioxidant defense parameters of the liver. However, treatment with *Z. multiflora* improved tissue integrity as well as antioxidant defense status and biochemical conditions of the liver.

**Conclusion:**

Administration of *Z. multiflora* extract has beneficial effects on the NaNO_2_-induced histological and functional toxicity in the liver.

## Introduction

Nitrate and nitrite ions are considered hazardous materials that are widespread in the environment. Despite its toxicity, nitrite is unavoidable in aqueous media because it is an essential intermediate in nitrogen metabolism. During denitrification process, nitrate converts into nitrite under anaerobic or anoxic conditions by the action of denitrifying microorganisms (Müller et al., 2018 ▶). Also, nitrite could be considered harmful to the human body if present in the dietary food intake (Karwowska and Kononiuk, 2020 ▶; Li and Liu, 2019 ▶). Industrially, alkali metal nitrites are produced by reaction of a mixture of nitrogen monoxide and nitrogen dioxide with the corresponding metal hydroxide solution. Nitrite is an intermediate product of the oxidation of ammonia to nitrate as well as an essential intermediate in the biological nitrogen cycle in the nature (Karwowska and Kononiuk, 2020 ▶). Sodium nitrite (NaNO_2_) has for decades been widely employed for preservation of meat products. It is also used as a color stabilizer, food flavor and antimicrobial agent in controlling the growth of *Clostridium botulinum* in the meat and fish processing industries (Alexander et al., 2009 ▶; Eyiler and Oztan, 2011 ▶; Adewale et al., 2019 ▶). Additionally, it acts as a vasodilator, bronchodilator and antidote for cyanide poisoning (Adewale et al., 2019 ▶; Kroupova et al., 2005 ▶). However, excessive intake of NaNO_2 _could be potentially life threatening because of its ability to induce oxidative DNA damage, inflammation, carcinogenicity and mutagenicity, resulting in organ damage (Fadda et al., 2018 ▶). Therefore, recent studies have indicated the potential adverse health effects of NaNO_2 _exposure in various tissues. Fadda et al. (2018) reported that a single sub-cutaneous dose of NaNO_2 _at 60 mg/kg caused cardiac injuries mediated by oxidative stress, inflammation, DNA damage and apoptosis (Fadda et al., 2018 ▶). In addition, it has been shown that sodium nitrite at a concentration of 5 mM had cytotoxic effects on isolated rat hepatocytes by reactive oxygen species (ROS) formation and lipid peroxidation mechanisms (Kiani et al., 2017 ▶). Furthermore, it has been found that NaNO_2_ at 60 mg/kg could exert hepatotoxicity through of C-reactive proteins, liver fat deposition, decreasing serum lipids, reducing antioxidants and increasing serum- hepatic transaminases and phosphatase (Adewale et al., 2019 ▶).

Increased attention has recently been paid to the herbal medicine and numerous medicinal plants have been considered by many to be used as an alternative for treatment and prevention of several chronic diseases (Chattopadhyay and Maurya, 2015 ▶; Padmavathi, 2013 ▶). *Zataria multiflora* (*Z. multiflora)* Boiss is species of the Labiatae family that is called “*Shirazi thyme”* in the traditional medicine and has different therapeutic properties. Recently, it was shown that oral administration of *Z. multiflora* at concentrations of 0.2 and 0.4 ml/kg body weight for 14 days had no adverse effect on liver and kidney integrity in the laboratory animals. Also, according to this study, *Z. multiflora* can to treat the acute toxoplasmosis in the mouse model due to its immuno-modulatory properties (Mahmoudvand et al., 2020 ▶). Therapeutic properties of *Z. multiflora* on the lung disorders of sulfur mustard-exposed individuals have been investigated (Khazdair et al., 2018 ▶). Another investigation recommended that *Z. multiflora* in combination to its constituent, carvacrol, is effective in the prevention of paraquat toxicity (Khazdair et al., 2018 ▶; Amin et al., 2020 ▶) as well as in alleviating asthma (Alavinezhad et al., 2017 ▶). Furthermore, on the basis of earlier works, *Z. multiflora* is rich in tannins, polymethoxy flavonoids, thymol and carvacrol compounds (Golkar et al., 2020 ▶; Mohebbati, 2018 ▶) and many of these ingredients especially thymol and carvacrol are responsible for its antioxidant, anti-inflammatory, antidiabetic and immunomodulatory properties (Khazdair et al., 2018 ▶; Khazdair et al., 2020 ▶; Khazdair et al., 2018 ▶; Khazdair et al., 2019 ▶; Mahmoodi et al., 2019 ▶ Rana et al., 2008 ▶). 

Due to the mechanism of NaNO_2_ toxicity and the ability of *Z. multiflora* to reduce inflammation and induce antioxidant defense, this study was performed to investigate the possibility of using this plant in alleviating hepatotoxicity after exposure to NaNO_2_ in rats.

## Materials and Methods

For extraction preparation, first, fresh *Z. multiflora* Boiss plant was obtained from local herbal market at Ilam Province (Ilam, Iran) during summer of 2019. Identification of plant was done by researchers from the Faculty of Agriculture, Ilam University and a voucher specimens (FAIU20205) was deposited at the Herbarium of that Faculty. Then, the leaves and stems of plant were dried in the shade. Three hundred grams of dried powder of plant was subjected to soxhlet extraction. Extraction process was completed by adding a combination of ethanol and water (ratio 7 to 3) at 45°C for 2 days. Then, the solvent of extract was evaporated using an evaporator at 60°C. The yield of extraction was 3.1% (w/w). Finally, the extract was dried at room temperature and stored at 4ºC.

**Figure 1:**
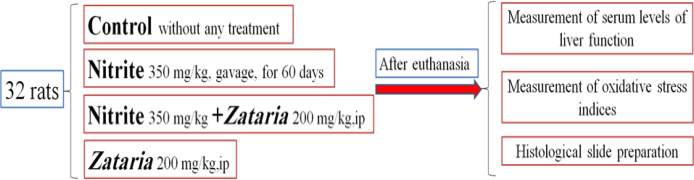
Experimental protocol

Thirty-two male Wistar rats, weighing 180-210 g, were randomly divided into control, nitrite, nitrite plus* Z. multiflora* and *Z. multiflora* groups. Control group (male rats received no treatment), nitrite group (rats treated with 350 mg/kg NaNO_2 _by gavage for 60 days), nitrite plus* Z. multiflora* group (rats treated with NaNO_2_ 350 mg/kg by gavage for 60 days and simultaneously received *Z. multiflora* extract at 200 mg/kg, ip) (Shebang et al., 2019) and *Z. multiflora* group (rats treated with *Z. multiflora* extract at 200 mg/kg, ip) (Figure 1). NaNO_2 _used in this research was obtained from (Merck Company, Germany). First, the appropriate concentration was determined and then dissolved in distilled water as a solvent. At the end of the experiment, all rats were anesthetized using ketamine (60 mg/kg, ip) and xylazine (10 mg/kg, ip), to collect blood samples from their heart. Separated serum was used for estimation of liver enzymes. Therefore, serum levels of alkaline phosphatase (ALP), alanine aminotransferase (ALT) and aspartate aminotransferase (AST) enzymes were measured using laboratory diagnostic kits from Pars Azmon Company. After opening the abdominal cavity, the liver samples were taken and stained with hematoxylin and eosin (H and E) for microscopic histological examinations. Liver tissues were homogenized in 0.1 M phosphate-buffered saline (1:5 w/v, neutral pH) and then centrifuged (4000 g for 20 min).

The supernatant separated was analyzed for oxidative stress parameters. Then, lipid peroxidation in the liver was measured in terms of malondialdehyde (MDA) levels, using the methods proposed by Ohkawa et al. (1979). MDA level was analyzed by the measurement of thio-barbituric acid reacting substances (TBARS) based on the colorimetric method and by a kit made by Navand lab. The level of superoxide dismutase (SOD) enzyme was measured based on inhibition of pyrogallol autoxidation reaction and using an *in vitro* assay kit made by Navand lab. The levels of glutathione peroxidase (GPX) enzyme were determined based on monitoring the oxidation of NADPH linked to oxidized glutathione (GSSG) reduction. Also, catalase (CAT) enzyme activity was measured using hydrogen peroxide as the substrate based on its peroxidase activity. The values are presented as mean±standard deviations (SD). Statistical analyses were done by SPSS software and using one-way analysis of variance (ANOVA) followed by Tukey's *post hoc* test, to compare the experimental groups. A p<0.05 was regarded as statistically significant.

## Results

**Figure 2:**
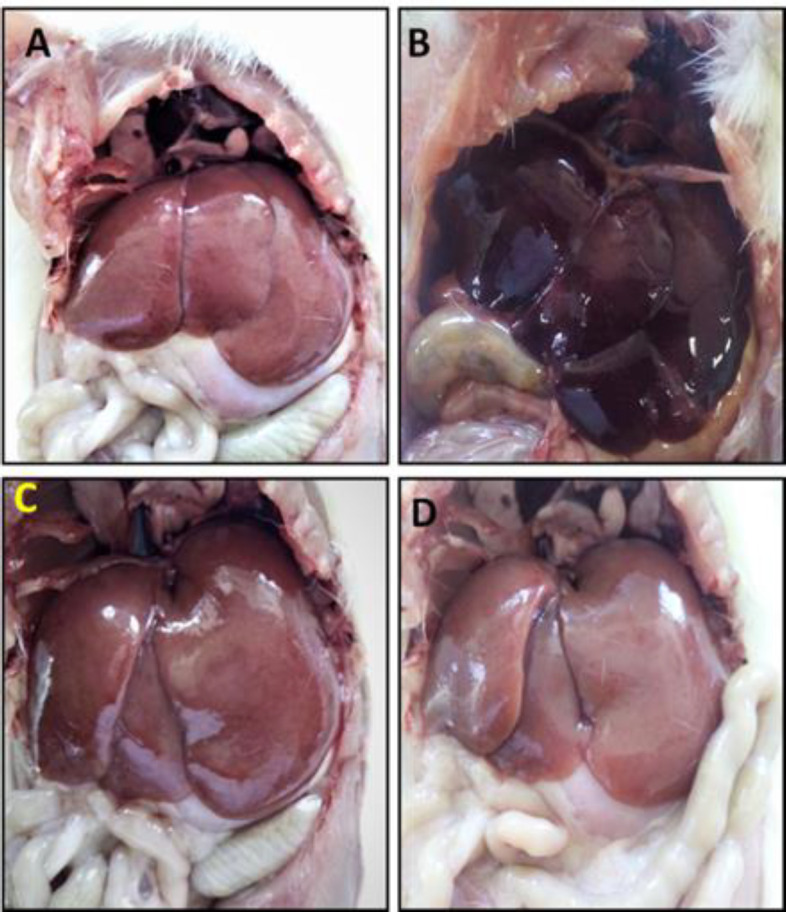
Anatomical study of the liver and the effect of hydroalcoholic extract of *Z. multiflora* in the liver of NaNO_2_-treated rats. A) The anatomy of control rats. B) The anatomy of liver in the NaNO_2_ treated rats. An increment in the bloody and dark appearance of liver is clear. C) Nitrite plus *Z. multiflora* group: The anatomy of liver in this animals showed fewer pathological changes and improved liver architecture. D) *Z. multiflora* group: The liver of these animals showed normal liver anatomical features

Anatomically, the livers of the control rats displayed normal color, size and consistency. Also, hepatic lobes with ordinary appearance and sharp margins were visible without any abnormal signs. In the NaNO_2_-treated animals, the color of the liver was clearly bloody and dark, and the overall size of the liver was larger compared to the control group. Liver sections of *Z. multiflora-*treated rats showed an improvement in the appearance, size and consistency as compared to the NaNO_2_ group and their structure was more similar to the control animals. No difference in macroscopic status of the liver was observed when comparing *Z. multiflora* with the healthy control group (Figure 2).

**Figure 3:**
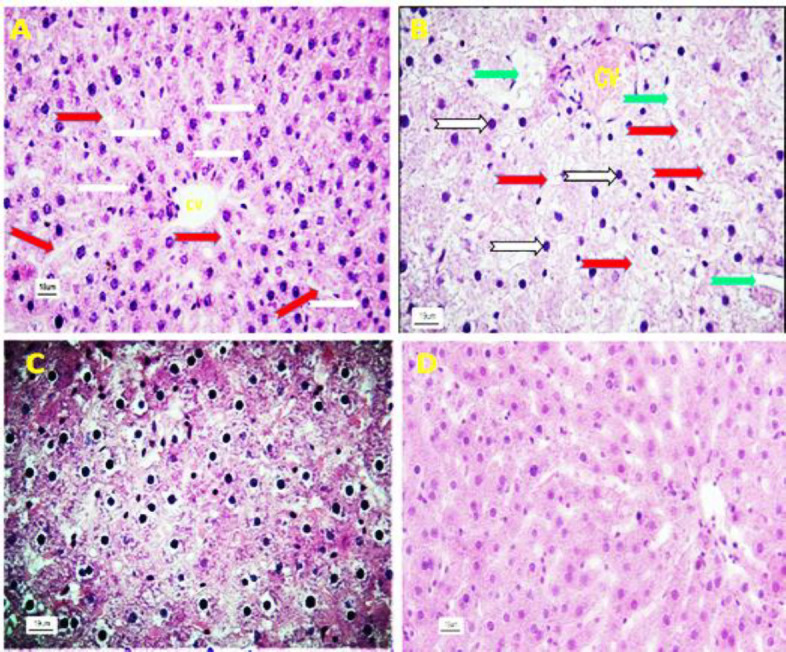
Histological study of liver tissue and the effect of hydroalcholic extract of *Z. multiflora* in liver tissues in NaNO_2_-treated rats. A) Control group showed no visible lesions. B) The microscopic structure of liver in the NaNO_2_-treated rats shows disruption in the order and arrangement of both hepatocytes and sinusoids. C) Nitrite plus *Z. multiflora* group: An improvement in the damaged liver tissue is remarkable. D) *Z. multiflora* group: The liver of these animals showed normal liver histology. (Red arrows: Sinusoids; White arrows: Hepatocytes; CV: Central vein; Green arrows: Dilated sinusoids; H&E stain at 400 x magnification)

**Figure 4:**
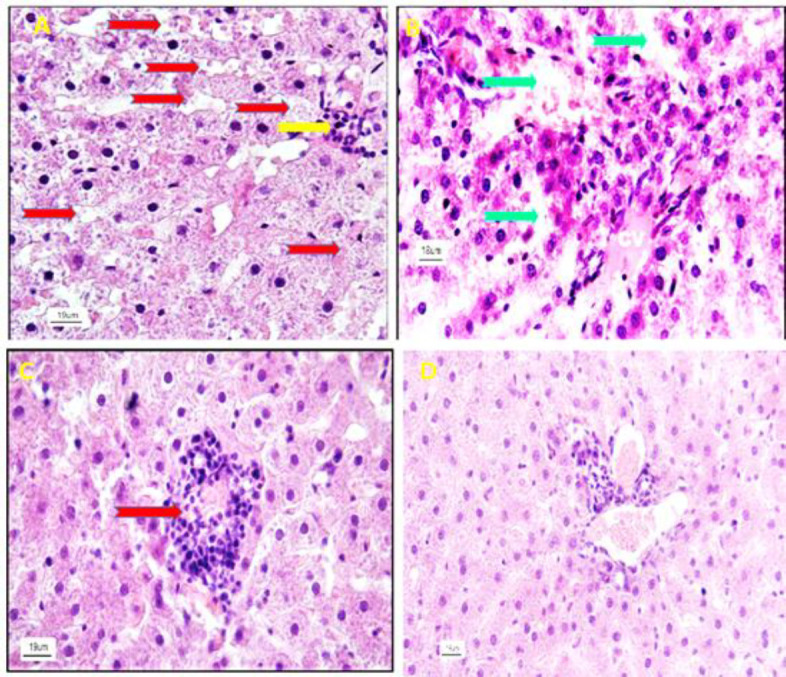
Histological study of the liver in the NaNO_2_-treated group. A) There is dilation and hyperemia in the sinusoids (red arrow) and infiltration of defense cells (yellow arrow) in the liver parenchyma. B) The presence of hyperemia in the central vein (CV), dilation of sinusoids (green arrow) and severe tissue inflammation are seen in the hepatic parenchyma. C) Invasion and focal accumulation of mononuclear cells (red arrow) are seen in the liver tissue. D) Infiltration of defense cells as well as hyperemia is seen in the portal hepatic area (H&E stain at 400 x magnification).

Histologically; liver sections of the control group did not show any changes, and the hepatocytes as well as sinusoids were normal with ordinary radial arrangements around the central vein. Also, hepatocytes of control rats showed a typical feature in the shape and size without any complications in their cytoplasm. No inflammatory cells infiltration was observed in the liver parenchyma (Figure 3). The photomicrographs of the liver sections of rats exposed to NaNO_2_ are presented in Figures 3 and 4. In these animals, a decrease in the cell density as well as disruption of the order and arrangement of both hepatocytes and sinusoids was observed. Generally, the structure of the liver cords was destroyed and natural discipline was not seen (Figure 3 and 4).

**Figure 5:**
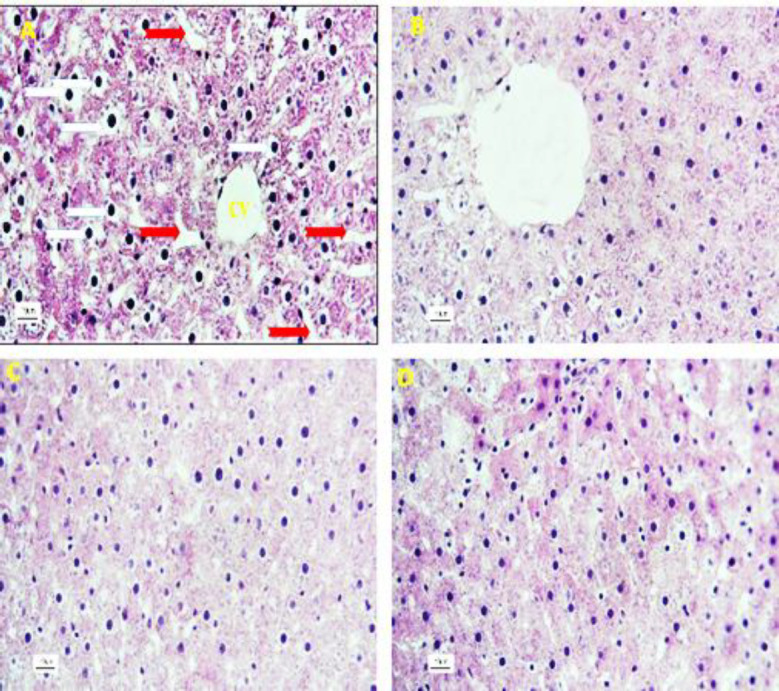
Histological study of liver tissue and the effect of hydroalcholic extract of *Z. multiflora* in liver tissues in NaNO_2_-treated rats. A) This part shows an increment in the cell density and an impartment in the hepatocytes shape and size (white arrow). Also, red arrow indicates slight dilation of the sinusoids. B) No hyperemia in the central vein is clear. C) Normal hepatocytes and sinusoids arrangement are seen in liver tissue. D) Reduced damage in liver tissue is remarkable (H&E stain at 400 x magnification)

In the nitrite plus *Z. multiflora* group, improvement of damaged liver tissue including increment of tissue density and correction of cellular and sinusoidal order were seen. However, in this group, leukocyte infiltration as well as a slight dilation in the sinusoids was seen (Figure 5). In the *Z. multiflora* group, no difference was observed in terms of tissue density and inflammation as compared to the control rats (Figure 3).

**Figure 6:**
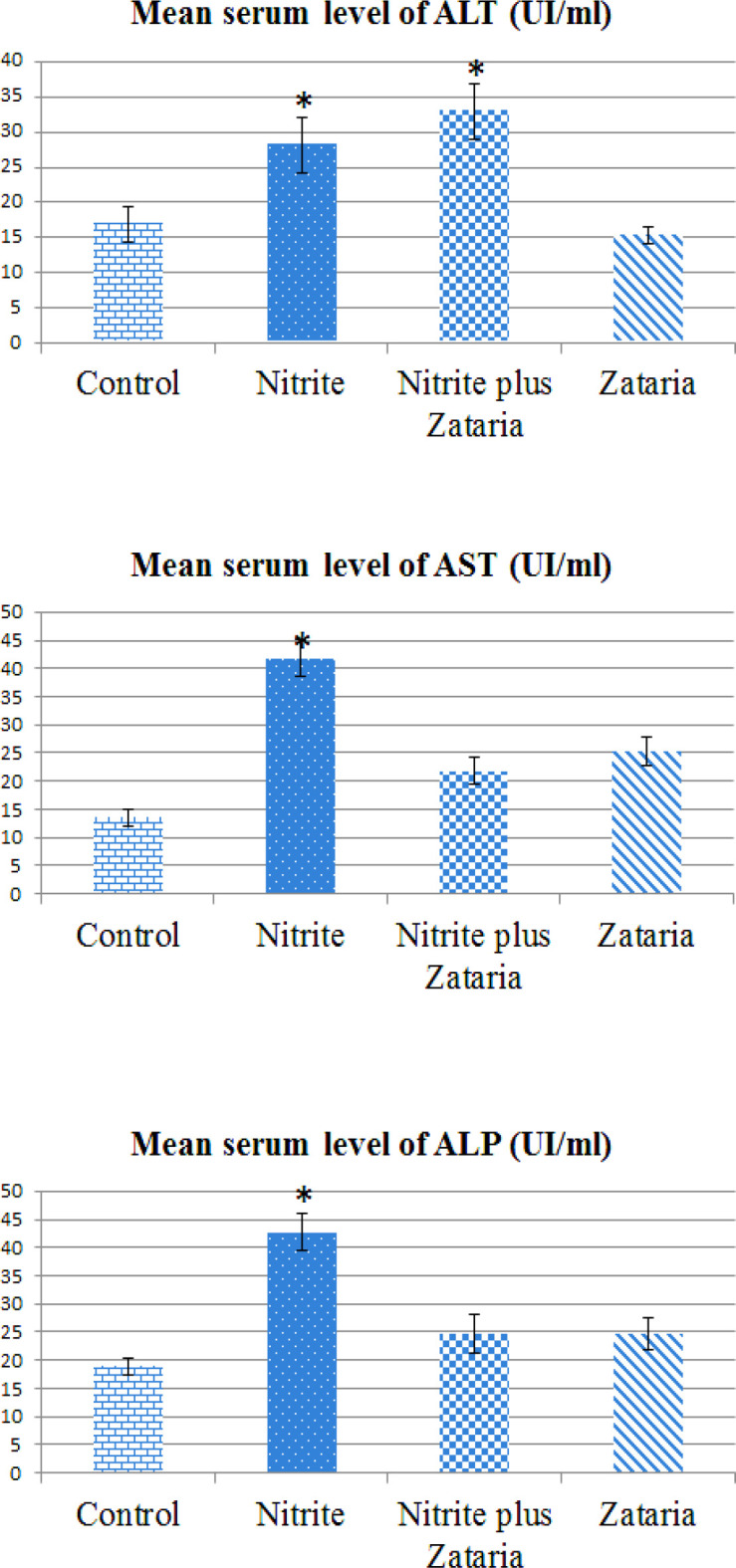
Mean serum level of hepatic enzymes in different groups. Data are presented as Mean±SD (n=8 in each group). (Abbreviations used: ALT: alanine aminotransferase; AST: aspartate aminotransferase; and ALP: alkaline phosphatase). *p<0.05 compared with the control group;

The effect of *Z. multiflora* administration on the liver’s function enzymes in the NaNO_2_-treated rats is shown in Figure 6. NaNO_2 _exposure significantly increased ALT, AST and ALP activities in comparison with the control group (p<0.05). Following *Z. multiflora* treatment, relative improvements in the hepatic enzymes function were seen. The serum levels of AST and ALP in the nitrite plus *Z. multiflora* group showed a significant decrease in comparison with the NaNO_2_-treated rats. But, their values always were higher than those of the control. The level of ALT activity was higher than that of the nitrite group, but this increment was not statistically significant. There was no significant difference in the ALT and ALP enzymes levels in the *Z. multiflora-*treated rats in comparison with the control group, but the AST activity was higher than that of the control; however, this increment was not significant (p<0.05, Figure 6).

**Figure 7:**
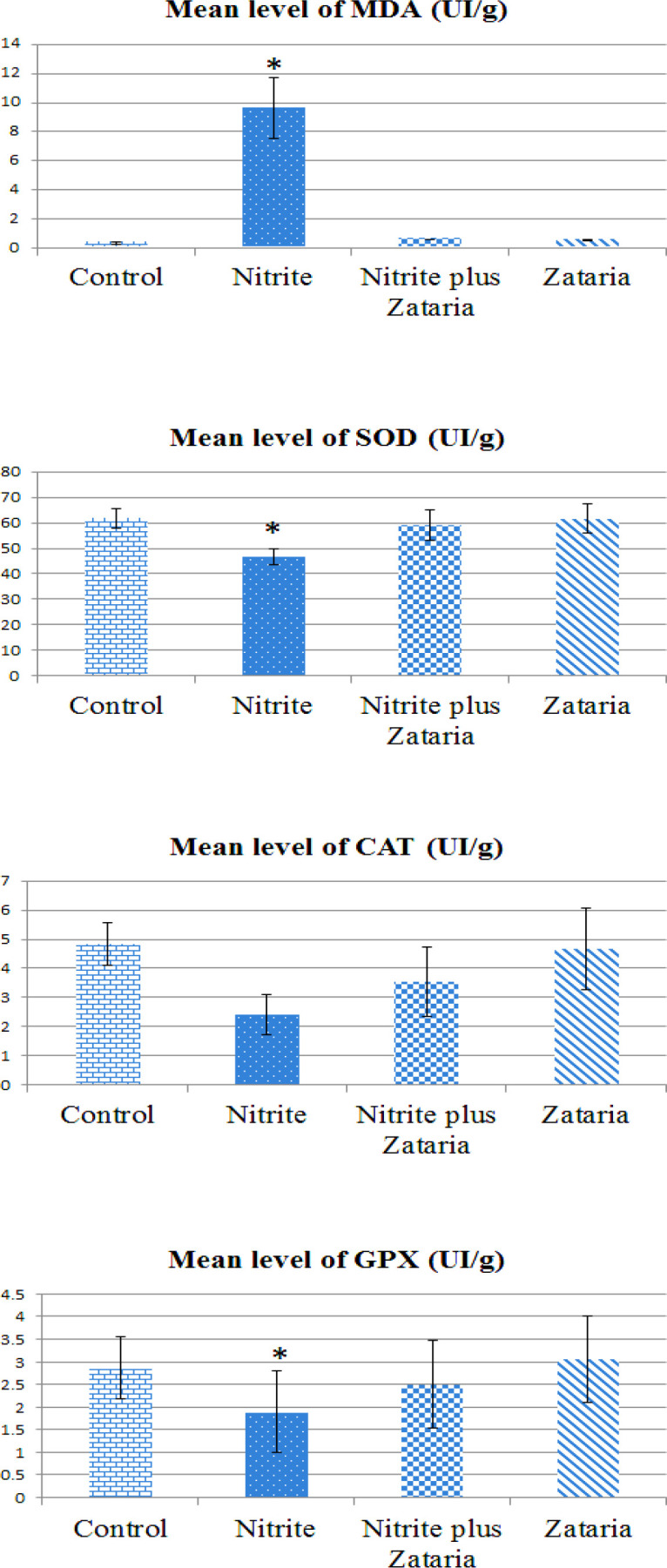
Mean level of malondialdehyde and antioxidant enzymes in different groups. Data are presented as Mean±SD (n=8 in each group). (Abbreviations used: MDA: malondialdehyde; SOD: superoxide dismutase; CAT: catalase; and GPX: glutathione peroxidase.) *p<0.05 compared with the control group

Figure 7 shows the effects of *Z. multiflora* on MDA, SOD, CAT and GPx activity in the NaNO_2_-treated rats. In terms of oxidative stress assay, there was a significant increase in the MDA level (p<0.05), as an indicator of lipid oxidation in the NaNO_2_-treated rats compared to the control,* Zataria *or nitrite plus *zataria* groups (Figure 7).

Also, the level of SOD enzyme reduced considerably (p<0.05) in the nitrite group, whereas the level of this enzyme increased markedly in the nitrite plus *zataria *group (p<0.05). There was no significant difference in the SOD levels between the *Z. multiflora-*treated rats and the control group. The level of CAT did not show significant differences among the various groups. Additionally, the level of GPX antioxidant enzyme in the nitrite group was significantly reduced (p<0.05) compared to the control group. Also, the activity of GPX in the *Z. multiflora-*treated rats was higher than that in the control group, but this difference was not significant (p<0.05, Figure 7).

## Discussion

In recent years, herbal medicines have gained special interest as alternative treatments for various disorders and chronic diseases (Chattopadhyay and Maurya, 2015 ▶; Padmavathi, 2013 ▶). Additionally, extensive use of nitrite and nitrate in the meat industries has increased worries about their detrimental effects on human health (Karwowska and Kononiuk, 2020 ▶). 

The liver is one of the unique organs involved in the detoxification of and metabolic activities on toxic exogenous substances that enter the body. Moreover, the hepatotoxicity induced by NaNO_2_ exposure has been proven in the earlier works (Mohamed Ali and Zeyadi, 2020 ▶; Adewale et al., 2019 ▶; Kiani et al., 2017 ▶). Hence, present research was performed to investigate the effect of NaNO_2_ on the liver integrity and as well as a possible ameliorative effect of *Z. multiflora*. In general, the results confirmed that treatment with sodium nitrite caused several structural complications in liver tissue and affected hepatic function. However, the use of *Z. multiflora* extract could attenuate the mentioned negative effects.

According to the results of macroscopic examination, consumption of NaNO_2 _caused severe enlargement and hyperemia of liver as presented by increased volume and size of the organ. According to a previous work, nitric oxide produced after sodium nitrite metabolism in the liver, can influence the smooth muscle of the vascular wall, cause more vasodilation and increase blood flow. This process could possibly lead to organ distention, hyperemia and black discoloration within the liver's tissues under anatomical examinations (Lundberg et al., 2008 ▶). On the other hand, considering the role of the liver in the metabolism of harmful exogenous chemicals, its increased size is possibly a compensatory hepatic cells response (Pek et al., 2021 ▶) to sodium nitrite as a toxic insult (Akhzari et al., 2019 ▶). 

In the present study, microscopic observations also confirmed macroscopic changes so that various structural abnormalities including sinusoidal disorders, parenchymal disorders, hyperemia and focal invasion of leucocytes were observed in sodium nitrite-treated animals. Regarding the possible mechanism of action of NaNO_2_, it has been reported that after increasing the tissue level of nitrite, the amount of nitric oxide radical increases. Upon reaction of the produced nitric oxide with superoxide radicals, peroxynitrite as a free radical is produced and later, it induces apoptosis or necrosis (Denicola and Radi, 2005 ▶; Li et al., 2004 ▶). It has also been reported that NaNO_2 _at a concentration of 5 mmol induces hepatoxicity in isolated rat hepatocytes by inducing oxidative stress (Kiani, 2017 ▶). Therefore, it could be concluded that in the present study, oral administration of NaNO_2 _increased the amount of nitric oxide radical. Dietary nitrite appeared to cause both free radicals and MDA formation and tissue damages. Increased levels of MDA in liver tissue act as another toxic agent to further increasing liver damage. The result is consistent with other researches (Marouf et al., 2011 ▶; Knowles et al., 1990 ▶). In the present study, increased MDA along with decreased antioxidant enzyme levels in the NaNO_2_-treated group indicate the inefficiency of the antioxidant defense system of the liver. These could be attributed to the hepatotoxic effect of NaNO_2_ that induce oxidative stress and inflammation. These findings are in agreement with the previous similar studies (Knowles et al., 1990 ▶; Marouf et al., 2011 ▶; Akhzari et al., 2019 ▶). In the present study, regarding the results of oxidative stress indices which are supported with histological changes, it seems that a possible mechanism involved in the NaNO_2 _hepatotoxicity is occurrence of oxidative stress. Oxidative stress occurs due to an imbalance in the presence of reactive oxygen species and the body's antioxidant activities and plays an axial role in activating various signaling pathways leading to tissue damage (Chatterjee, 2016 ▶). Because free radicals directly attack cell membrane phospholipids, the level of lipid peroxidation is an important indicator of oxidative stress (Mehdipour et al., 2013 ▶). The development of oxidative stress in the liver following treatment with NaNO_2 _is due to the induction of lipid peroxidation. When nitrites react with amines in the stomach, nitrosamines and free radicals are formed. Nitrosamine increases lipid peroxidation and thus, impairs the maintenance of tissue integrity and hepatocyte´s function (Akhzari et al., 2019 ▶). 

Present findings demonstrated significant elevation in the liver enzymes activity in blood of NaNO_2_-treated rats. ALT and AST are located in the cytoplasm and mitochondria of hepatocytes and their amounts maybe elevated in the severe liver damages such as hepatitis, cirrhosis and ischemia (Yap and Choon, 2010 ▶). In line with this, it has been reported that *Z. multiflora* extract at a concentration of 800 ppm in drinking water given for consecutive 7 days, can ameliorate the serum levels of liver function enzymes in halothane-exposed rats (Sakhaee et al., 2011 ▶). These results are consistent with the findings of the present study.

In general, both macroscopic and microscopic observations as well as functional enzymatic of liver and oxidative stress indices demonstrated that *Z. multiflora* administration can significantly prevent much of sodium nitrite hepatotoxicity. The exact mechanism(s) underlying the hepatoprotective effects of* Z. multiflora *is not clear, but it has been suggested that the plant contains effective compounds such as flavonoids, thymol and caracrol, which are responsible for its hepatoprotective properties (Blumenthal, 2000 ▶; Golkar et al., 2020 ▶; Mohebbati, 2018 ▶). 

Consistent with this hypothesis, there are several reports that suggested that antioxidative effects of *Z. multiflora *play an axial role in the hepatoprotective activity (Khazdair et al., 2018 ▶; Hajihashemi et al., 2015 ▶; Mohebbati, 2018 ▶; Sakhaee et al., 2011 ▶). One of the major findings of the current study was severe tissue inflammation and mononuclear defense cells infiltration in the different areas of liver of NaNO_2_-treated animals. On the other hand, *Z. multiflora* administration did reduce the severity of hepatic lesions. In accordance with this result, a previous study demonstrated the anti-inflammatory activity of this medicinal plant against sulfur mustard-induced lung inflammation in veterans (Khazdair et al., 2020 ▶). Also, according to a previous work, carvacol as a main component of *Z. multiflora *extract exerts anti-inflammatory and antioxidant properties and it can improve peak expiratory flow value in the sulfur mustard-exposed patients (Khazdair et al., 2018 ▶). Additionally, Sharififar et al. (2011) ▶ reported that treatment with concentrations of 100, 200 and 400 μl / kg of *Z. multiflora* orally for 10 days has antioxidant effects and can eliminate free radicals and their harmful effects in a dose-dependent manner (Sharififar et al., 2011 ▶). Furthermore, Khazdair et al. (2018) ▶ demonstrated the pharmacological effects of *Z. multiflora* and its constituents focusing on their anti-inflammatory, antioxidant, and immunomodulatory effects. Thus, it is concluded that another possible mechanism for hepatoprotective effects of* Z. multiflora *is due to its anti-inflammatory properties. Accordingly, another study reported radical scavenging properties of essential oils from *Z. multiflora*; the authors stated this plant can be used as a natural antioxidant in the therapy of oxidative damage that tends to accompany some inflammatory conditions. They attributed the radical scavenging effect of *Z. multiflora* to its phenolic content (Kavoosi et al., 2012 ▶).

The present study had several limitations such as not examining long-term exposure to NaNO_2 _and its possible carcinogenicity. Other limitations were the lack of electron microscopy studies to find the exact location and organelles of hepatocytes which are involved in NaNO_2 _toxicity and the therapeutic effects of *Z. multiflora* extract. In general, the results showed that NaNO_2 _treatment causes many destructive changes in liver tissue as well as enzymes levels. The NaNO_2_-induced hepatotoxicity was ameliorated by administration of *Z. multiflora* extract.

Ameliorative effects of *Z. multiflora* against NaNO_2_ induced hepatotoxicity may be attributed to its anti-oxidative and anti-inflammatory properties. Therefore, *Z. multiflora* can be mentioned as a treatment strategy to improve the effects of NaNO_2_ on liver injury and function. Further studies involving electron microscopy investigations need to be carried out to determine the exact mechanism underlying protective effect of *Z. multiflora* against NaNO_2_ hepatotoxicity.

## Conflicts of interest

The authors have declared that there is no conflict of interest.
